# Supplementary material for: Maintenance of specificity in sympatric host-specific fig/wasp pollination mutualisms
Source: PeerJ. 2022 Aug 11;10:e13897. doi: 10.7717/peerj.13897 (PMC9375967; doi:10.7717/peerj.13897)
Supplement: Supplemental Information 1 — a compounds with their identity confirmed by mass spectrometry analysis; b compounds with their identity confirmed by comparison of Kovats retention index with the NIST chemistry Web Book (http://webbook.nist.gov) and literature; c compounds with their identity confirmed by comparison of MS and retention time with laboratory standards (Sigma-Aldrich, USA). [file peerj-10-13897-s001.docx]

Table S1

| Kovats RI | Compound | *Ficus auriculata* Male | | | *F. auriculata* Female | | | *F. hainanensis* Male | | | *F. hainanensis* Female | | |
| --- | --- | --- | --- | --- | --- | --- | --- | --- | --- | --- | --- | --- | --- |
|  |  | Occ | Mean | SD | Occ | Mean | SD | Occ | Mean | SD | Occ | Mean | SD |
|  |  | n | mean | sd | n | mean | sd | n | mean | sd | n | mean | sd |
| 887 | 2-Heptanone ^a,b^ | 2 | 2.09 | 2.22 | 3 | 0.82 | 0.41 |  |  |  |  |  |  |
| 937 | α-Pinene ^a,b^ | 1 | 0.08 |  | 1 | 0.37 |  | 2 | 0.07 | 0.01 | 2 | 0.05 | 0.03 |
| 974 | Sabinene ^a,b^ |  |  |  | 2 | 0.48 | 0.45 | 2 | 0.21 | 0.19 | 3 | 0.40 | 0.50 |
| 1024 | Limonene ^a,b^ | 2 | 0.49 | 0.26 |  | 0.00 | 0.00 |  |  |  |  |  |  |
| 1035 | 1-Hexanol ^a,b^ | 2 | 1.24 | 1.14 |  | 0.00 | 0.00 |  |  |  |  |  |  |
| 1305 | Eucalyptol ^a,b^ | 1 | 2.05 |  | 2 | 0.37 | 0.31 |  |  |  |  |  |  |
| 1040 | β-cis-Ocimene ^a,b,c^ | 1 | 0.11 |  | 3 | 0.18 | 0.04 |  |  |  |  |  |  |
| 1049 | β-trans-Ocimene ^a,b,c^ | 4 | 2.25 | 3.14 | 3 | 5.31 | 2.80 | 2 | 2.43 | 2.78 | 3 | 2.73 | 1.05 |
| 1065 | Linalool oxide ^a,b^ | 0 |  |  | 2 | 0.21 | 0.05 |  |  |  | 2 | 0.05 | 0.06 |
| 1096 | Linalool ^a,b,c^ | 2 | 0.52 | 0.37 | 2 | 0.04 | 0.07 |  |  |  | 1 | 0.06 |  |
| 1221 | Cyclohexane ^a,b^ | 2 | 2.13 | 3.21 | 1 | 0.12 |  | 2 | 0.07 | 0.01 | 3 | 0.21 | 0.21 |
| 1350 | Maaliene ^a,b^ | 1 | 1.06 |  |  | 0.00 | 0.00 |  |  |  |  |  |  |
| 1135 | 2,6-Dimethyl-1,3,5,7-octatetraene ^a,b^ |  |  |  | 3 | 1.08 | 0.67 |  |  |  |  |  |  |
| 1338 | δ-Elemene ^a,b^ | 4 | 1.07 | 0.22 | 3 | 1.68 | 1.11 | 3 | 0.15 | 0.07 | 3 | 0.69 | 0.46 |
| 1351 | Sesquichamene ^a,b^ | 4 | 0.43 | 0.31 |  | 0.00 | 0.00 | 3 | 0.03 | 0.01 |  |  |  |
| 1351 | α-Cubenene ^a,b^ | 1 | 0.62 |  | 3 | 0.45 | 0.39 |  |  |  | 3 | 0.02 | 0.02 |
| 1357 | Longipinene ^a,b^ | 2 | 0.40 | 0.01 | 2 | 0.10 | 0.06 | 3 | 0.07 | 0.01 | 3 | 0.43 | 0.43 |
| 1367 | Cyclosativene ^a,b^ |  |  |  | 3 | 0.87 | 0.53 |  |  |  |  |  |  |
| 1374 | Cyclocitral ^a,b^ |  |  |  | 2 | 0.11 | 0.14 |  |  |  |  |  |  |
| 1375 | Ylangene ^a,b^ | 2 | 2.62 | 2.67 | 3 | 5.01 | 4.43 | 3 | 0.61 | 0.36 | 3 | 2.30 | 1.79 |
| 1377 | Isoledene ^a,b^ | 3 | 1.18 | 0.75 | 3 | 1.58 | 1.23 | 3 | 1.52 | 1.27 | 3 | 2.65 | 1.16 |
| 1380 | α-Copaene ^a,b^ | 4 | 5.04 | 6.46 | 3 | 1.06 | 0.76 | 3 | 9.02 | 0.53 | 3 | 6.32 | 1.89 |
| 1381 | Longicyclene ^a,b^ | 1 | 1.42 |  |  | 0.00 | 0.00 |  |  |  |  |  |  |
| 1382 | Tridecane ^a,b^ |  |  |  | 1 | 0.05 |  |  |  |  |  |  |  |
| 1383 | Unknown 1 |  |  |  | 3 | 0.09 | 0.05 |  |  |  |  |  |  |
| 1384 | α-Funebrene ^a,b^ | 3 | 3.21 | 3.89 | 3 | 0.55 | 0.38 | 3 | 3.60 | 1.21 | 3 | 5.88 | 1.90 |
| 1387 | πBourbonene ^a,b^ |  |  |  |  |  |  | 1 | 0.04 |  |  |  |  |
| 1390 | β-Cubebene ^a,b^ | 1 | 1.08 |  | 3 | 0.92 | 1.01 | 3 | 0.39 | 0.19 | 3 | 2.05 | 0.65 |
| 1391 | Unknown 2 |  |  |  | 3 | 0.40 | 0.22 |  |  |  |  |  |  |
| 1392 | Unknown 3 |  |  |  | 2 | 0.34 | 0.15 |  |  |  |  |  |  |
| 1393 | β-Elemene ^a,b^ | 4 | 1.29 | 0.69 | 3 | 0.79 | 0.51 |  |  |  | 3 | 0.42 | 0.38 |
| 1395 | Unknown 4 |  |  |  | 3 | 0.20 | 0.11 |  |  |  |  |  |  |
| 1400 | Tetradecane ^a,b, c^ | 2 | 0.82 | 0.22 |  | 0.00 | 0.00 |  |  |  |  |  |  |
| 1402 | Longifolene ^a,b^ |  |  |  |  |  |  | 3 | 1.15 | 0.81 | 1 | 0.35 |  |
| 1405 | α-Cedrene ^a,b^ | 4 | 2.00 | 0.71 | 2 | 1.02 | 0.78 | 3 | 7.29 | 0.23 | 3 | 9.75 | 3.18 |
| 1406 | cis-α-Bergamotene ^a,b^ |  |  |  | 3 | 4.62 | 5.31 |  |  |  |  |  |  |
| 1409 | α-Gurgujene ^a,b,c^ | 4 | 6.62 | 6.12 | 3 | 8.66 | 4.67 |  |  |  | 2 | 0.59 | 0.58 |
| 1417 | β-Funebrene ^a,b^ | 4 | 14.15 | 6.58 | 3 | 9.62 | 2.90 | 3 | 28.96 | 5.40 | 3 | 24.70 | 5.68 |
| 1423 | β-Caryophellene ^a,b,c^ | 4 | 3.10 | 0.24 |  | 0.00 | 0.00 |  |  |  |  |  |  |
| 1424 | β-Cedrene ^a,b^ |  |  |  | 3 | 3.64 | 2.88 | 3 | 5.29 | 2.36 | 3 | 9.01 | 4.24 |
| 1434 | β-Gurjurene ^a,b^ | 4 | 0.65 | 0.45 |  | 0.34 | 0.35 | 3 | 1.35 | 0.56 | 3 | 2.07 | 0.20 |
| 1435 | γ-Elemene ^a,b^ |  |  |  |  |  |  | 3 | 0.79 | 0.45 | 3 | 0.62 | 0.34 |
| 1437 | α-Bergamotene ^a,b^ |  |  |  | 2 | 2.38 | 1.31 |  |  |  |  |  |  |
| 1438 | Aromadendrene ^a,b^ | 4 | 0.48 | 0.22 |  | 0.00 | 0.00 |  |  |  |  |  |  |
| 1439 | Unknown 5 |  |  |  |  |  |  | 3 | 1.11 | 0.59 | 1 | 0.91 |  |
| 1440 | α-Guaiaene ^a,b^ |  |  |  |  |  |  | 3 | 9.46 | 3.19 | 3 | 1.58 | 0.44 |
| 1441 | Aromadendrene ^a,b^ |  |  |  |  |  |  | 3 | 13.63 | 5.63 | 3 | 8.12 | 2.18 |
| 1447 | (Z)-β-Farnesene ^a,b^ | 2 | 0.25 | 0.14 | 3 | 0.42 | 0.38 |  |  |  |  |  |  |
| 1450 | Himachalene ^a,b^ | 4 | 3.81 | 1.38 | 3 | 2.17 | 2.50 | 3 | 1.13 | 0.75 | 3 | 3.08 | 1.21 |
| 1451 | Seychellene ^a,b^ | 1 | 3.12 |  | 3 | 2.77 | 1.74 |  |  |  |  |  |  |
| 1454 | Alloaromadendrene ^a,b^ |  |  |  | 1 | 2.08 |  |  |  |  |  |  |  |
| 1457 | (E)-β-Farnesene ^a,b^ | 4 | 8.21 | 5.70 | 3 | 5.09 | 2.50 |  |  |  | 3 | 2.12 | 1.28 |
| 1463 | α-Patchoulene ^a,b^ | 4 | 12.07 | 6.56 | 3 | 4.78 | 3.23 |  |  |  |  |  |  |
| 1465 | Unknown 6 |  |  |  |  |  |  | 3 | 2.80 | 2.01 | 3 | 1.05 | 0.51 |
| 1467 | Cadinene ^a,b^ |  |  |  | 3 | 1.71 | 1.28 |  |  |  |  |  |  |
| 1478 | Chamigrene ^a,b^ | 3 | 2.99 | 0.38 | 3 | 1.85 | 0.96 |  |  |  | 1 | 0.75 |  |
| 1476 | γ-Gurjunene ^a,b^ | 1 | 2.72 |  |  | 0.00 | 0.00 | 1 | 0.22 |  |  |  |  |
| 1477 | Γ-Muurolene ^a,b^ | 4 | 1.39 | 1.42 |  | 0.00 | 0.00 | 3 | 5.69 | 1.45 | 3 | 3.31 | 1.92 |
| 1484 | β-Cubebene ^a,b^ | 3 | 2.79 | 1.43 | 3 | 0.45 | 0.27 |  |  |  | 2 | 0.38 | 0.23 |
| 1486 | Germacrene D ^a,b^ | 2 | 0.89 | 0.27 | 3 | 0.81 | 0.89 |  |  |  |  |  |  |
| 1494 | (Z,E)-α-Farnesene ^a,b^ | 1 | 3.54 |  | 2 | 1.22 | 0.72 |  |  |  | 1 | 0.06 |  |
| 1496 | Elixene ^a,b^ | 3 | 3.11 | 0.33 | 3 | 2.66 | 1.05 |  |  |  |  |  |  |
| 1502 | α-Muurolene ^a,b^ |  |  |  | 3 | 0.94 | 0.28 | 1 | 0.17 |  |  |  |  |
| 1503 | α-Chamigrene ^a,b^ |  |  |  |  |  |  | 3 | 0.19 | 0.10 | 2 | 0.54 | 0.50 |
| 1509 | (E,E)-α-Farnesene ^a,b^ |  |  |  | 3 | 9.75 | 15.25 |  |  |  | 2 | 0.06 | 0.04 |
| 1520 | Calamenene ^a,b^ |  |  |  |  |  |  | 3 | 0.46 | 0.18 |  |  |  |
| 1520 | β-Cadinene ^a,b^ | 2 | 4.15 | 3.54 | 3 | 0.39 | 0.04 |  |  |  |  |  |  |
| 1523 | Germacrene B ^a,b^ | 2 | 0.45 | 0.41 |  | 0.00 | 0.00 |  |  |  |  |  |  |
| 1525 | Sesquiphellandrene ^a,b^ | 2 | 0.88 | 0.25 | 3 | 3.59 | 1.55 | 3 | 0.29 | 0.11 | 3 | 2.89 | 0.92 |
| 1559 | Viridiflorol ^a,b^ | 2 | 1.59 | 0.06 |  | 0.23 |  |  |  |  | 3 | 0.74 | 0.15 |
| 1583 | Spathulenol ^a,b^ |  |  |  | 3 | 0.38 | 0.20 |  |  |  | 3 | 0.46 | 0.29 |
| 1598 | (+)-Ledol ^a,b^ |  |  |  | 2 | 2.17 | 2.14 |  |  |  |  |  |  |
| 1600 | Hexadecane ^a,b,c^ | 2 | 0.48 | 0.05 |  | 0.00 | 0.00 |  |  |  |  |  |  |
| 1627 | Unknown 7 | 2 | 0.28 | 0.02 |  | 0.00 | 0.00 | 3 | 0.30 | 0.16 | 3 | 0.29 | 0.26 |
| 1675 | Unknown 8 | 2 | 0.25 | 0.07 |  | 0.00 | 0.00 | 3 | 0.81 | 0.79 | 3 | 0.62 | 0.18 |
| 1690 | Longiverbenone ^a,b^ | 3 | 2.56 | 1.86 | 3 | 2.09 | 1.58 |  |  |  |  |  |  |
| 1871 | Phthalic acid ^a,b^ | 2 | 2.56 | 1.62 |  |  |  |  |  |  |  |  |  |
