# Supplementary material for: Maintenance of specificity in sympatric host-specific fig/wasp pollination mutualisms
Source: PeerJ. 2022 Aug 11;10:e13897. doi: 10.7717/peerj.13897 (PMC9375967; doi:10.7717/peerj.13897)
Supplement: Supplemental Information 6 [file peerj-10-13897-s006.docx]

**Statistical result**

1. **Pollinator bioassay**

1.1 result of Fig 4

> chisq.test(F_au)

Chi-squared test for given probabilities

data: F_au

X-squared = 30, df = 1, p-value = 4.32e-08

> chisq.test(F_ha)

Chi-squared test for given probabilities

data: F_ha

X-squared = 1.2, df = 1, p-value = 0.2733

> chisq.test(F_cross)

Chi-squared test for given probabilities

data: F_cross

X-squared = 5.1212, df = 1, p-value = 0.02364

**2. recorded time between F.auriculata and F. hainanensis**

> wilcox_test(time~treatment, data = bio_choice_time)

Asymptotic Wilcoxon-Mann-Whitney Test

data: time by treatment (F_au, F_ha)

Z = -0.078346, p-value = 0.9376

alternative hypothesis: true mu is not equal to 0

> wilcox_test(time~treatment, data = bio_choice_time)

Asymptotic Wilcoxon-Mann-Whitney Test

data: time by treatment (F_au, F_ha)

Z = -2.6407, p-value = 0.008274

alternative hypothesis: true mu is not equal to 0

**3. PERMANOVA on sex and group**

library(vegan)

vocsa<-read.csv("F:/Desktop/case/auriculata/vocsa_sex.csv", header = TRUE)

View(vocsa)

vocsag<- read.csv("F:/Desktop/case/auriculata/vocsa_gsex.csv", header = TRUE)

View(vocsag)

ad1<-adonis(vocsa~ Sex,data = vocsag,permutations = 999,method="bray")

pairwise.adonis <-function(x,factors, sim.method, p.adjust.m)

{

co = as.matrix(combn(unique(factors),2))

pairs = c()

F.Model =c()

R2 = c()

p.value = c()

ad = adonis(x[factors %in%c(as.character(co[1,elem]),as.character(co[2,elem])),] ~

factors[factors %in%c(as.character(co[1,elem]),as.character(co[2,elem]))] , method =sim.method);

pairs =c(pairs,paste(co[1,elem],'vs',co[2,elem]));

F.Model =c(F.Model,ad$aov.tab[1,4]);

R2 = c(R2,ad$aov.tab[1,5]);

p.value = c(p.value,ad$aov.tab[1,6])

}

p.adjusted =p.adjust(p.value,method=p.adjust.m)

pairw.res = data.frame(pairs,F.Model,R2,p.value,p.adjusted)

return(pairw.res)

}

pairwise.adonis(vocsa, vocsag$Sex, sim.method="bray", p.adjust.m= "bonferroni")

3.1 F. auriculata

> pairwise.adonis(vocsa, vocsag$Sex, sim.method="bray", p.adjust.m= "bonferroni")

Set of permutations < 'minperm'. Generating entire set.

pairs F.Model R2 p.value p.adjusted

1 Male vs Female 2.676781 0.3486854 0.062 0.062

3.2 F. hainanensis

> pairwise.adonis(vocsa, vocsag$Sex, sim.method="bray", p.adjust.m= "bonferroni")

'nperm' >= set of all permutations: complete enumeration.

Set of permutations < 'minperm'. Generating entire set.

pairs F.Model R2 p.value p.adjusted

1 Male vs Female 3.947555 0.4967006 0.1 0.1

3.3 comparison on F. auriculata and F. hainanensis

pairwise.adonis(vocsa, vocsag$Group, sim.method="bray", p.adjust.m= "bonferroni")

pairs F.Model R2 p.value p.adjusted

1 a vs h 11.29675 0.5066546 0.002 0.002

3.4 NMDS analysis

library(permute)

library(lattice)

library(vegan)

library(MASS)

sem <- read.csv("F:/Desktop/case/Pei/New_vocs.csv", header = TRUE)

sem=na.omit(sem)

fix(sem)

attach(sem)

sem.x<-sem[,2:78]

sem.mds<-metaMDS(sem.x, "bray",autotransform = TRUE)

plt<-plot(sem.mds, display="sites", type= "n",main=paste("Stress=", round(sem.mds$stress,3)))

points (plt$sites[1:7,], pch=15, cex=2,bg=sem$color, col="red")

points (plt$sites[8:13,], pch=16, cex=2,bg=sem$color,col="blue")

with(sem, ordispider(sem.mds, Tree, col = "gray5"))

with(sem, ordihull(sem.mds, Tree, col = "gray5",lty = 2))

legend(0,0.82, c("F. auriculata", "F. hainanensis"),pch = c(15,16),col ="black", cex=1.5, text.font = 3,

x.intersp = 0.25,y.intersp = 0.45,

text.col = "black", box.col = 0)

**4. mean length of style and ovipositor**

**4.1 F. auriculata**

> wilcox_test(Au_style~sex_au, data = bio_style)

Asymptotic Wilcoxon-Mann-Whitney Test

data: Au_style by sex_au (1, 2)

Z = -25.534, p-value < 2.2e-16

alternative hypothesis: true mu is not equal to 0

**4.2 F. hainanensis**

> wilcox_test(Ha_style~sex_ha, data = bio_style)

Asymptotic Wilcoxon-Mann-Whitney Test

data: Ha_style by sex_ha (1, 2)

Z = -30.292, p-value < 2.2e-16

alternative hypothesis: true mu is not equal to 0

**4.3 male fig between F. auriculata and F. hainanensis**

> wilcox_test(Male_style~Ficus, data = bio_style)

Asymptotic Wilcoxon-Mann-Whitney Test

data: Male_style by Ficus (1, 2)

Z = 9.2945, p-value < 2.2e-16

alternative hypothesis: true mu is not equal to 0

**4.4 Ovipositor length between Ce and Csh**

> wilcox_test(Ovipositor~species, data = bio_style)

Asymptotic Wilcoxon-Mann-Whitney Test

data: Ovipositor by species (1, 2)

Z = 9.4864, p-value < 2.2e-16

alternative hypothesis: true mu is not equal to 0

**5 Consequences of introduction of *C. emarginatus***

5.1 offspring number

> wilcox_test(flower~Ce_in_off, data=bio_introduction)

Asymptotic Wilcoxon-Mann-Whitney Test

data: flower by Ce_in_off (1, 2)

Z = -4.4895, p-value = 7.139e-06

alternative hypothesis: true mu is not equal to 0

**5.2 wasp size**

$means

head std r LCL UCL Min Max Q25 Q50 Q75

1 0.4815104 0.04530041 77 0.4721133 0.4909075 0.3846 0.6410 0.4615 0.4872 0.5128

2 0.4622826 0.03956738 69 0.4523557 0.4722096 0.3590 0.5385 0.4359 0.4615 0.4872

3 0.4646789 0.04081110 114 0.4569559 0.4724020 0.3590 0.5641 0.4359 0.4615 0.4872

$comparison

NULL

$groups

head groups

1 0.4815104 a

3 0.4646789 b

2 0.4622826 b

attr(,"class")

[1] "group"

> TurkeyHSD(bio_avo)

Error in TurkeyHSD(bio_avo) : could not find function "TurkeyHSD"

> TukeyHSD(bio_avo)

Tukey multiple comparisons of means

95% family-wise confidence level

Fit: aov(formula = head ~ Ficus, data = bio_wasp)

$Ficus

diff lwr upr p adj

2-1 -0.019227781 -0.03559133 -0.002864236 0.0165128

3-1 -0.016831442 -0.03139239 -0.002270490 0.0187820

3-2 0.002396339 -0.01266000 0.017452680 0.9253671

$means

Thorax std r LCL UCL Min Max Q25 Q50 Q75

1 0.5867325 0.04903974 77 0.5739642 0.5995007 0.4615 0.6667 0.5641 0.5897 0.615400

2 0.5518348 0.04496751 69 0.5383467 0.5653229 0.4359 0.6410 0.5385 0.5641 0.589700

3 0.5521693 0.06729027 114 0.5416757 0.5626629 0.3846 0.6410 0.5128 0.5641 0.608975

$comparison

NULL

$groups

Thorax groups

1 0.5867325 a

3 0.5521693 b

2 0.5518348 b

attr(,"class")

[1] "group"

> TukeyHSD(bio_avo1)

Tukey multiple comparisons of means

95% family-wise confidence level

Fit: aov(formula = Thorax ~ Ficus, data = bio_wasp)

$Ficus

diff lwr upr p adj

2-1 -0.0348976849 -0.05713146 -0.01266391 0.0007684

3-1 -0.0345631693 -0.05434769 -0.01477865 0.0001519

3-2 0.0003345156 -0.02012311 0.02079214 0.9991811

$groups

Ovipositor groups

1 1.151515 a

2 1.069917 b

3 1.034405 c

attr(,"class")

[1] "group"

> TukeyHSD(bio_avo2)

Tukey multiple comparisons of means

95% family-wise confidence level

Fit: aov(formula = Ovipositor ~ Ficus, data = bio_wasp)

$Ficus

diff lwr upr p adj

2-1 -0.08159776 -0.1178066 -0.045388939 0.0000007

3-1 -0.11710989 -0.1493300 -0.084889797 0.0000000

3-2 -0.03551213 -0.0688284 -0.002195853 0.0335781
