# Supplementary material for: Maintenance of specificity in sympatric host-specific fig/wasp pollination mutualisms
Source: PeerJ. 2022 Aug 11;10:e13897. doi: 10.7717/peerj.13897 (PMC9375967; doi:10.7717/peerj.13897)
Supplement: Supplemental Information 7 [file peerj-10-13897-s007.docx]

Statistical result

1. Style-length comparison among figs and trees
2. F. auriculata

style length of male among figs

> kruskal.test(Style_length$Male_Au_style~Style_length$Fruit_m_Au, data = Style_length)

Kruskal-Wallis rank sum test

data: Style_length$Male_Au_style by Style_length$Fruit_m_Au

Kruskal-Wallis chi-squared = 635.99, df = 31, p-value < 2.2e-16

Among trees

> kruskal.test(Style_length$Male_Au_style~Style_length$Male_tree_au, data = Style_length)

Kruskal-Wallis rank sum test

data: Style_length$Male_Au_style by Style_length$Male_tree_au

Kruskal-Wallis chi-squared = 584.53, df = 3, p-value < 2.2e-16

style length of female among figs

> kruskal.test(Style_length$Female_Au_style~Style_length$Fruit_f_au, data = Style_length)

Kruskal-Wallis rank sum test

data: Style_length$Female_Au_style by Style_length$Fruit_f_au

Kruskal-Wallis chi-squared = 597.48, df = 29, p-value < 2.2e-16

> kruskal.test(Style_length$Male_Au_style~Style_length$Female_tree_au, data = Style_length)

Kruskal-Wallis rank sum test

data: Style_length$Male_Au_style by Style_length$Female_tree_au

Kruskal-Wallis chi-squared = 531.46, df = 2, p-value < 2.2e-16

1. F.hainanensis

> kruskal.test(Style_length$Male_hai_style~Style_length$Fruit_m_hai, data = Style_length)

Kruskal-Wallis rank sum test

data: Style_length$Male_hai_style by Style_length$Fruit_m_hai

Kruskal-Wallis chi-squared = 68.808, df = 29, p-value = 4.406e-05

> kruskal.test(Style_length$Male_hai_style~Style_length$Male_tree_hai, data = Style_length)

Kruskal-Wallis rank sum test

data: Style_length$Male_hai_style by Style_length$Male_tree_hai

Kruskal-Wallis chi-squared = 16.087, df = 2, p-value = 0.0003211

> kruskal.test(Style_length$Female_hai_style~Style_length$Fruit_f_au, data = Style_length)

Kruskal-Wallis rank sum test

data: Style_length$Female_hai_style by Style_length$Fruit_f_au

Kruskal-Wallis chi-squared = 597.18, df = 29, p-value < 2.2e-16

> kruskal.test(Style_length$Female_hai_style~Style_length$Female_tree_hai, data = Style_length)

Kruskal-Wallis rank sum test

data: Style_length$Female_hai_style by Style_length$Female_tree_hai

Kruskal-Wallis chi-squared = 549.64, df = 2, p-value < 2.2e-16

1. Offspring-size comparison among figs and trees

*Ceratosolen emarginatus*

*Among figs*

> kruskal.test(Ce_headwidth~Ce_fig, data = Body_siz)

Kruskal-Wallis rank sum test

data: Ce_headwidth by Ce_fig

Kruskal-Wallis chi-squared = 21.204, df = 14, p-value = 0.09651

> kruskal.test(Ce_thoraxwidth~Ce_fig, data = Body_siz)

Kruskal-Wallis rank sum test

data: Ce_thoraxwidth by Ce_fig

Kruskal-Wallis chi-squared = 10.706, df = 14, p-value = 0.7089

> kruskal.test(Ce.Ovilength~Ce_fig, data = Body_siz)

Kruskal-Wallis rank sum test

data: Ce.Ovilength by Ce_fig

Kruskal-Wallis chi-squared = 22.266, df = 14, p-value = 0.07332

Among trees

> kruskal.test(Ce_headwidth~Ce_tree, data = Body_siz)

Kruskal-Wallis rank sum test

data: Ce_headwidth by Ce_tree

Kruskal-Wallis chi-squared = 5.1641, df = 2, p-value = 0.07562

> kruskal.test(Ce_thoraxwidth~Ce_tree, data = Body_siz)

Kruskal-Wallis rank sum test

data: Ce_thoraxwidth by Ce_tree

Kruskal-Wallis chi-squared = 1.3399, df = 2, p-value = 0.5117

> kruskal.test(Ce.Ovilength~Ce_tree, data = Body_siz)

Kruskal-Wallis rank sum test

data: Ce.Ovilength by Ce_tree

Kruskal-Wallis chi-squared = 1.3452, df = 2, p-value = 0.5104

*Ceratosolen* sp.

*Among figs*

> kruskal.test(Ceh_Headwidth~Ceh_fig, data = Body_siz)

Kruskal-Wallis rank sum test

data: Ceh_Headwidth by Ceh_fig

Kruskal-Wallis chi-squared = 13.365, df = 10, p-value = 0.204

> kruskal.test(Ceh_Thoraxwidth~Ceh_fig, data = Body_siz)

Kruskal-Wallis rank sum test

data: Ceh_Thoraxwidth by Ceh_fig

Kruskal-Wallis chi-squared = 7.7914, df = 10, p-value = 0.6492

> kruskal.test(Ceh_Ovilength~Ceh_fig, data = Body_siz)

Kruskal-Wallis rank sum test

data: Ceh_Ovilength by Ceh_fig

Kruskal-Wallis chi-squared = 5.5267, df = 10, p-value = 0.8533

*Among trees*

> kruskal.test(Ceh_Headwidth~Ceh_tree, data = Body_siz)

Kruskal-Wallis rank sum test

data: Ceh_Headwidth by Ceh_tree

Kruskal-Wallis chi-squared = 5.3184, df = 2, p-value = 0.07001

> kruskal.test(Ceh_Thoraxwidth~Ceh_tree, data = Body_siz)

Kruskal-Wallis rank sum test

data: Ceh_Thoraxwidth by Ceh_tree

Kruskal-Wallis chi-squared = 0.72915, df = 2, p-value = 0.6945

> kruskal.test(Ceh_Ovilength~Ceh_tree, data = Body_siz)

Kruskal-Wallis rank sum test

data: Ceh_Ovilength by Ceh_tree

Kruskal-Wallis chi-squared = 3.6767, df = 2, p-value = 0.1591
